# Meniscus maturation in the swine model: changes occurring along with anterior to posterior and medial to lateral aspect during growth

**DOI:** 10.1111/jcmm.12367

**Published:** 2014-09-12

**Authors:** Alessia Di Giancamillo, Daniela Deponti, Alessandro Addis, Cinzia Domeneghini, Giuseppe M Peretti

**Affiliations:** a IRCCS Istituto Ortopedico GaleazziMilan, Italy; b CRABCC, Biotechnology Research Center for Cardiothoracic ApplicationsRivolta d'Adda (CR), Italy; c Department of Health, Animal Science and Food Safety, Università degli Studi di MilanoMilano, Italy; d Department of Biomedical Sciences for Health, Università degli Studi di MilanoMilano, Italy

**Keywords:** meniscus, collagen fibres, glycosaminoglycans, fibrochondrocytes

## Abstract

The meniscus plays important roles in knee function and mechanics and is characterized by a heterogeneous matrix composition. The changes in meniscus vascularization observed during growth suggest that the tissue-specific composition may be the result of a maturation process. This study has the aim to characterize the structural and biochemical variations that occur in the swine meniscus with age. To this purpose, menisci were collected from young and adult pigs and divided into different zones. In study 1, both lateral and medial menisci were divided into the anterior horn, the body and the posterior horn for the evaluation of glycosaminoglycans (GAGs), collagen 1 and 2 content. In study 2, the menisci were sectioned into the inner, the intermediate and the outer zones to determine the variations in the cell phenotype along with the inner–outer direction, through gene expression analysis. According to the results, the swine meniscus is characterized by an increasing enrichment in the cartilaginous component with age, with an increasing deposition in the anterior horn (GAGs and collagen 2; *P* < 0.01 both); moreover, this cartilaginous matrix strongly increases in the inner avascular and intermediate zone, as a consequence of a specific differentiation of meniscal cells towards a cartilaginous phenotype (collagen 2, *P* < 0.01). The obtained data add new information on the changes that accompany meniscus maturation, suggesting a specific response of meniscal cells to the regional mechanical stimuli in the knee joint.

## Introduction

The knee meniscus is a fibro-cartilaginous tissue with a semi-lunar shape interposed between the femoral condyles and the tibial plateau, having functions of load bearing, shock absorption, joint stability, joint lubrication and allowing for a precise congruity between the femoral condyles and the tibial plateau of the knee joint 1–3.

The meniscus displays great regional variation in its extracellular matrix components. Its periphery is highly fibrous, abundant in cells and collagen 1, while the inner portion of the tissue resembles hyaline cartilage with fewer cells, a higher proteoglycan content and the presence of collagen 2 4–6. The outer portion of the meniscus, the so called ‘red’ zone is highly vascularized, while the inner meniscus is devoid of blood vessels 7–10, and consequently named the ‘white’ zone; an intermediate zone is also described, the ‘red-white’ zone, localized between the outer and the inner ones, whose morpho-functional characters are intermediate, too. All these features are typical of a ‘mature’ meniscus, typical of adulthood, and they are the results of several changes in vascularity, cellularity and matrix molecular organization that occur during the growth of this highly specialized structure. After birth, the meniscus continues to grow and refines its collagen architecture, and the cells start to vary in their synthetic profiles regionally 5,11–13, while the vascularity decreases in the inner meniscus 14. All these changes involving meniscus morphology and matrix composition during its maturation are the result of several biomechanical stimuli within the knee joint: different biomechanical forces can induce different cell responses and synthetic activity that are at the base of the complexity of the meniscus 7,15–18; on the other hand, this heterogeneity allows the meniscus to mediate specific biomechanical functions in the different areas, such as in the body and in the horns: the variations in tensile stiffness and strength appear to be related to local differences in the collagen fibre ultrastructure and fibre bundle direction 19,20; moreover, the viscoelastic response of the meniscus to load depends on the collagen 21 and proteoglycan contents of this tissue 22: different compressive aggregate modulus has been observed in the body with respect to the two horns 19 suggesting the presence of a different matrix composition in these regions.

A better understanding of the regional variations in the meniscus matrix composition and the changes occurring throughout meniscus maturation would lead to a deeper knowledge of the events and signals regulating meniscus growth, leading to the development of new strategies for meniscus engineering.

The purpose of this study was to characterize the morphology, the matrix composition and the cell phenotype of the meniscus in a pig model to highlight the changes that occur during its maturation from youth to adulthood, both in the medial and lateral menisci. In particular, different zones were compared in the young and adult swine meniscus, developing two studies, which are intimately linked: study 1 was focused on the anterior horn, the central body and the posterior horn; these regions were compared by morphological, biochemical and immunohistochemical analysis; study 2 was focused on the inner (the ‘white’ zone), intermediate (the ‘red-white’ zone) and outer (the ‘red’ zone) areas that were compared by gene expression analysis to determine the phenotypical changes that accompany the decrease in vascularization during meniscus growth. The obtained results will possibly be used for the construction of a swine model that will be translated to the comprehension of maturation phases of meniscus in humans.

## Materials and methods

### Study design

**Figure 1:**
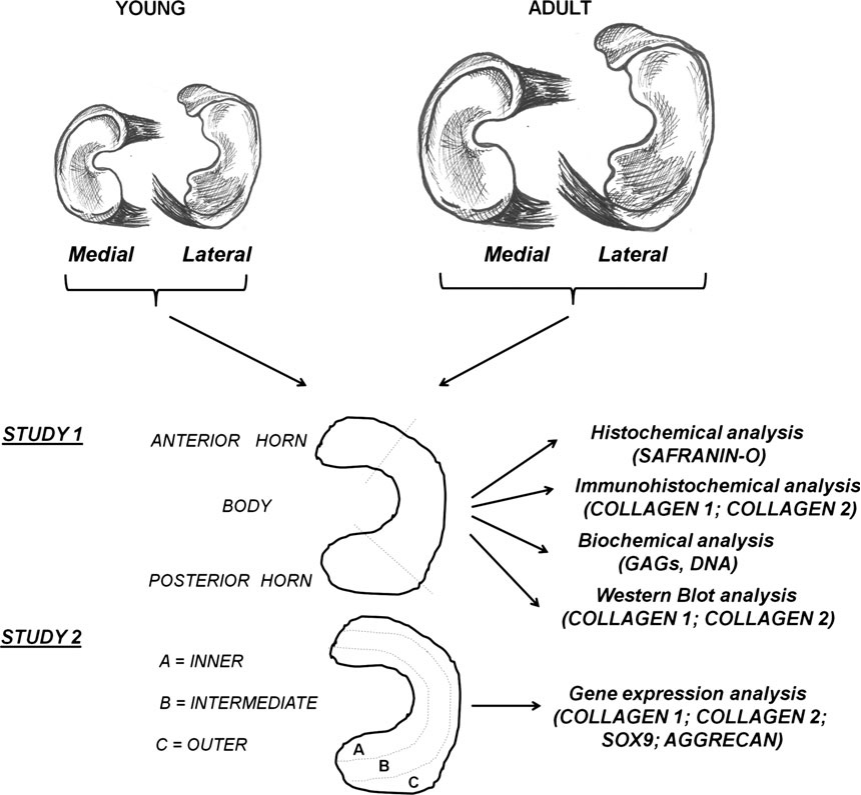
Experimental design. Medial and lateral menisci were harvested from young and adult pigs. In study 1, the swine menisci were sectioned into the anterior horns, the bodies and the posterior horns and processed for SAFRANIN-O staining, collagen 1 and 2 immunofluorescent staining, DNA and GAGs quantification, collagen 1 and 2 quantification by Western blot analysis. In study 2, the swine menisci were sectioned into the inner, the intermediate and the external zones to determine the expression levels of collagen 1, collagen 2, Sox9 and aggrecan by real-time PCR.

Experimental design is depicted in Figure1. The knee joints of young (1-month old) and adult (∼7-month old) female pigs (Landras x Large white, average weight 10–12 kg and 75–90 kg, respectively) were obtained from a local slaughterhouse and they were dissected to isolate the tibia and to remove menisci. Capsular tissue and ligaments were removed and the menisci were differently sectioned according to the purpose of the study. In study 1, both of them were transversally sectioned into the three parts that corresponded to their anterior horn, the central body and the posterior horn. All samples were analysed by: (*i*) standard GAGs histochemistry with SAFRANIN-0 staining for describing both the menisci structure and the GAGs deposition in the matrix; (*ii*) double immunofluorescence for collagen 1 and 2 with the aim of identifying single and double immunoreactive structures; (*iii*) western blot analysis to quantify the deposition of collagen 1 and 2 in the matrix and (*iv*) biochemical assays for the quantification of the DNA and glycosaminoglycans (GAGs) contents. In study 2, both the menisci from young and adult pigs were sectioned into the inner, intermediate and outer areas, to determine the differences in the cell phenotype by real-time PCR.

### Micro-anatomical analyses (Study 1)

Samples for the histochemical and immunofluorescence analyses were fixed in 10% (v/v) phosphate-buffered formaldehyde. The samples were then dehydrated in a graded 50% (v/v), 70% (v/v), 95% (v/v) and 100% (v/v) ethanol series, embedded in paraffin and transversally cut into 4-μm-thick consecutive sections. For each experimental group (young and adult, lateral and medial menisci: anterior horn, body and posterior horn), four specimens were analysed (total nr = 48).

#### Histochemistry: SAFRANIN-O staining

The different meniscus sections were stained with SAFRANIN-O using a standard staining protocol for the evaluation of the meniscus structure and evaluation of GAGs deposition in the matrix. The samples were analysed with an Olympus BX51 light microscope (Olympus, Opera Zerbo, Milan Italy) equipped with a digital camera, and final magnifications were calculated.

#### Double immunofluorescence

This procedure was utilized with the aim of revealing the possible co-localization of the two different types of collagen. After rehydration, heat-induced antigen retrieval was performed as previously described 23. After washing three times in PBS (pH 7.4), sections were incubated with the first-step primary antiserum, 1:50 collagen 1 (Abcam, Cambridge, UK) for 24 hrs at 18–20°C, then washed in PBS, and subsequently treated with the Avidin–Biotin blocking kit solution (Vector Laboratories Inc., Burlingame, CA USA). The sections were then washed in PBS for 10 min. and incubated with a solution of goat biotinylated anti-rabbit IgG (Vector Laboratories Inc.), 10 μg/ml in Tris-buffered saline (TBS) for 1 hr at 18–20°C. After rinsing twice in PBS, the sections were treated with Fluorescein–Avidin D (Vector Laboratories Inc.), 10 μg/ml in NaHCO_3_, 0.1 M, pH 8.5, 0.15 M NaCl for 1 hr at 18–20°C. For the second step of the double immunofluorescence procedure, sections were treated in a 2% hyaluronidase solution at room temperature for 30 min. The slides were subsequently treated with 1:50 anti-collagen 2 antiserum (Chondrex Inc., Redmond, WA USA). Sections were rinsed in TBS for 10 min. and incubated with 10 μg/ml goat biotinylated antimouse IgG (Vector Laboratories Inc.) for 1 hr at 18–20°C. The sections were then washed twice in PBS, and treated with Rhodamine–Avidin D (Vector Laboratories Inc.), 10 μg/ml in NaHCO_3_, 0.1 M, pH 8.5, with 0.15 M NaCl for 1 hr at 18–20°C. Finally, slides with tissue sections were embedded in Vectashield Mounting Medium (Vector Laboratories Inc.) and observed using a Confocal Laser Scanning Microscope (FluoView FV300; Olympus). The immunofluororeactive structures were excited using Argon/Helio–Neon–Green lasers with excitation and barrier filters set for fluorescein and rhodamine. Images containing superimposition of fluorescence were obtained by sequentially acquiring the image slice of each laser excitation or channel. In double immunofluorescence experiment, the absence of cross-reactivity with the secondary antibody was verified by omitting the primary antibody during the first incubation step.

### Biochemical analysis (Study 1)

According to the similarities observed between medial and lateral menisci by previous micro-anatomical analysis, medial and lateral meniscal samples were pooled for the biochemical analysis. For each experimental group (young and adult: anterior horn, body and posterior horn), 12 specimens were processed (total nr = 72). The samples for biochemical evaluation were digested in papain (Sigma-Aldrich, Milan, Italy) for 16–24 hrs at 60°C; the digestion solution was composed of 125 μg/ml of papain (Sigma-Aldrich) in 100 mM sodium phosphate, 10 mM sodium EDTA (Sigma-Aldrich), 10 mM cysteine hydrochloride (Sigma-Aldrich), 5 mM EDTA adjusted to pH 6.5 and brought to 100 ml of solution with distilled water. The digested samples were stored at −80°C until analyses. Aliquots of the papain digests were assayed separately for proteoglycan and DNA contents. Proteoglycan content was estimated by quantifying the amount of sulphated glycosaminoglycans using the 1,9-dimethylmethylene (DMB) blue dye binding assay (Polysciences Inc., Washington, PA, USA) and a microplate reader (wavelength: 540 nm). The standard curve for the analysis was generated using bovine trachea chondroitin sulphate A (Sigma-Aldrich). DNA content was evaluated with the Quant-iT Picogreen dsDNA Assay Kit (Molecular Probes, Inc., Eugene, OR, USA) and a fluorescence microplate reader and standard fluorescein wavelengths (excitation 485 nm, emission 538 nm, cut-off 530 nm). The standard curve for the analysis was generated using bacteriophage lambda DNA supplied with the kit.

### Protein extraction and Western blot (Study 1)

According to the similarities observed between medial and lateral menisci by previous micro-anatomical analysis, medial and lateral meniscal samples were pooled for the Western blot analysis. For each experimental group (young and adult: anterior horn, body and posterior horn), eight specimens were processed (total nr = 48). The samples were pulverised for 2 min. at 3000 oscillations/min. in a liquid nitrogen cooled dismembrator (Mikro-Dismembrator, Sartorius Stedim, Muggiò, Italy); then they were homogenized in a buffer containing 50 mM Tris-HCl, 150 mM NaCl, 0.1% SDS, 0.5% sodium deoxycholate, 1% NP40, pH 7.4, supplemented with protease inhibitor cocktail (Euroclone, Pero, Italy) and centrifuged at 13000 g at 4°C for 10 min. to discard cellular debris. Protein concentration in the extracts was determined using BCA protein assay (Euroclone). After addition of 0.05% bromophenol blue, 10% glycerol, and 2% β-mercaptoethanol, 50 μg of each sample was boiled and loaded onto 6% SDS–polyacrylamide gels. After electrophoresis, polypeptides were electrophoretically transferred to nitrocellulose filters (Sigma-Aldrich); the membranes were incubated with 5% non-fat milk for 1 hr at room temperature to block the non-specific sites and then probed for 2 hrs at room temperature using the following antibodies (Abs): anti-collagen 2 (1:1000; no. ab3092; Abcam); anti-collagen 1 (1:500; no. NB600-408; Novus Biologicals, Littleton, CO, USA) and anti-GAPDH (clone GAPDH-71.1; Sigma-Aldrich). The membranes were then washed and incubated for 1 hr at room temperature with HRP-labelled secondary antibodies (1:5000; Bio-Rad, Hercules, CA, USA). The blots were developed using a chemiluminescent substrate (WESTAR Nova 2011, Cyanagen, Bologna, Italy).

### RNA extraction and reverse transcription (Study 2)

According to the similarities observed between medial and lateral menisci by previous micro-anatomical analysis, medial and lateral meniscal samples were pooled for RNA extraction and gene expression analysis. For each experimental group (young and adult: inner, intermediate and outer areas), eight specimens were analysed (total nr = 48). The samples were pulverised for 2 min. at 3000 oscillations/min. in a liquid nitrogen cooled dismembrator (Mikro-Dismembrator, Sartorius Stedim). A 1 ml aliquot of Qiazol (Qiagen Sciences, Germantown, MD, USA) was added to the powdered samples in microcentrifuge tubes; after 10 min., 200 μl of chloroform was added to each sample prior the centrifugation at 12,000 × g for 15 min. at 4°C. RNA was extracted from the aqueous phase using a commercially available RNA purification kit (RNeasy Mini Kit, Qiagen). Digestion of DNA was carried out using a commercially available kit (RNase-Free DNase Set, Qiagen). RNA was stored at −80°C prior to reverse transcription.

The purified RNAs were measured (ND-1000 spectrophotometer Nanodrop Technologies, Wilmington, DE, USA) and then 600 ng RNA were used to prepare cDNA using ImPromII Reverse Transcription System according to the manufacturer's instructions (Promega Italia, Milan, Italy). Briefly, RNA was added to reverse transcription mixture that was composed of random hexamer primers, water, reaction buffer, MgCl_2_, dNTPmix, Ribonuclease inhibitor and reverse transcriptase. The reaction was carry out at 42°C for 1 hr. cDNAs were stored at −20°C prior to relative quantification of gene expression.

### Real-time PCR (Study 2)

Real-time PCR was performed with the iQ SYBR green Supermix (Bio-Rad Laboratories Inc.). cDNA was amplified using specific primers 23 at a concentration of 0.4 μM. After activating the DNA polymerase by incubation for 2 min. at 95°C, 40 cycles of amplification were carried out. After PCR, the baseline subtraction method was used to determine the threshold cycle. Data were expressed as ratio between target mRNA and housekeeping gene β-2Microglobulin (β2-M) mRNA.

### Statistical analyses

Statistical analysis of the data (biochemical results, Western blot and Real-time PCR: young, adult, and young *versus* adult) was performed with the general linear model of the SAS (version 8.1; Cary, NC, USA). The individual meniscal samples were considered to be the experimental unit of all response variables. The data were presented as least squared means ± SEM. Differences between means were considered significant at *P* < 0.05.

## Results

### Study 1

#### Micro-anatomical analyses

**Figure 2:**
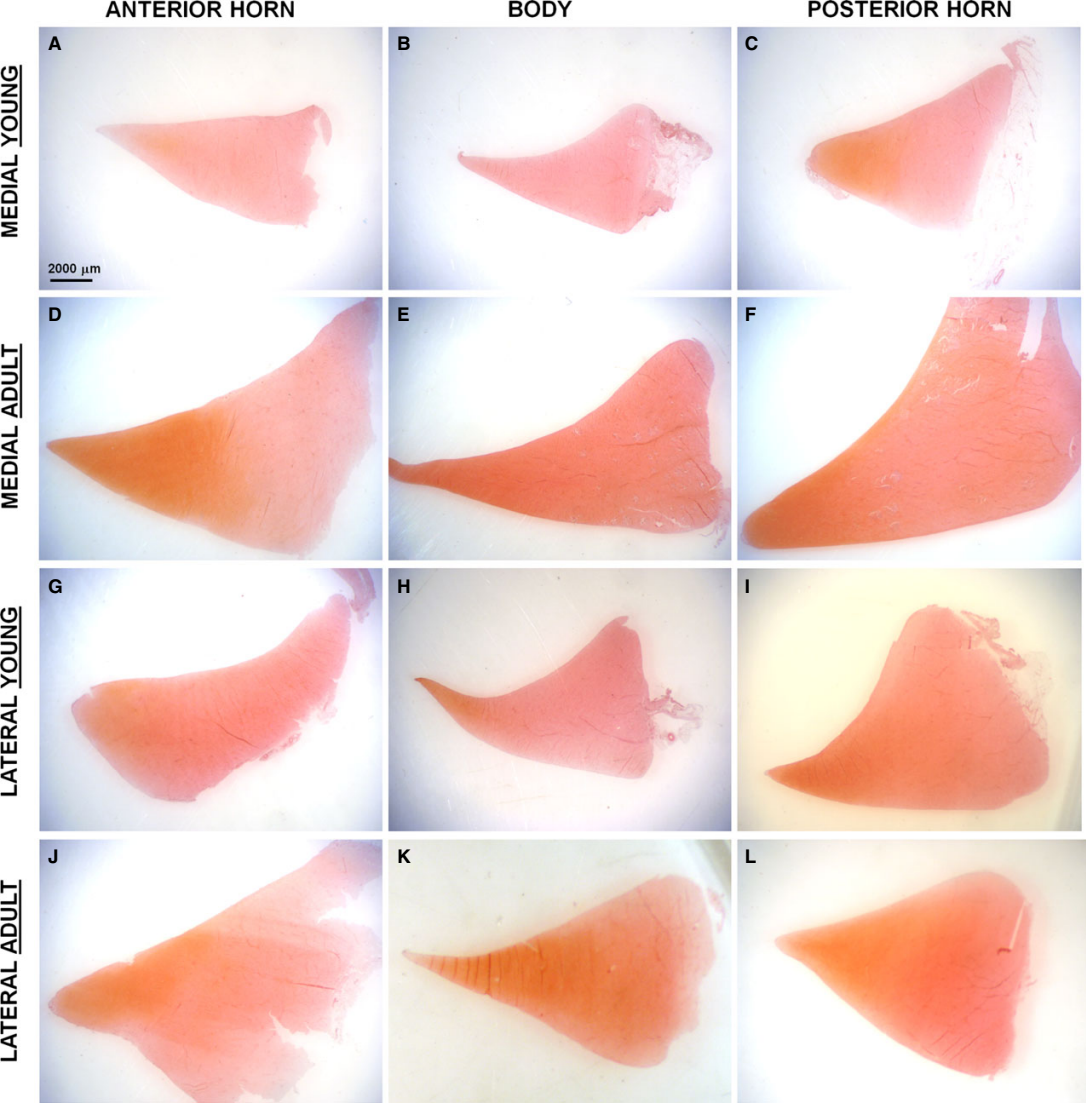
SAFRANIN-O staining on traversal sections of the meniscus. Scale bar 2000 μm. (**A**) Anterior horn, medial young meniscus; (**B**) Body, medial young meniscus; (**C**) Posterior horn, medial young meniscus; (**D**) Anterior horn, medial adult meniscus; (**E**) Body, medial adult meniscus; (**F**) Posterior horn, medial adult meniscus; (**G**) Anterior horn, lateral young meniscus; (**H**) Body, lateral young meniscus; (**I**) Posterior horn, lateral young meniscus; (**J**) Anterior horn, lateral adult meniscus; (**K**) Body, lateral adult meniscus; (**L**) Posterior horn, lateral adult meniscus.

*SAFRANIN-O staining*. The results of SAFRANIN-0 histochemistry is depicted in Figure2.

In the young model, both the medial and lateral menisci showed a posterior horn having a stronger SAFRANIN-0 positivity in the matrix with respect to the anterior horn and the body (Fig.2c and i). In the medial meniscus, the SAFRANIN 0 reactivity was very scarce in the anterior horn and the central body (Fig.2a and b).

In the adult model, the medial and lateral menisci showed similar SAFRANIN 0 positivity, and the positive staining was generally characterized by an higher level in the inner zone of the meniscus that gradually decreases moving to the outer region of the meniscus; both the medial and lateral menisci were characterized by a stronger GAGs deposition in the two horns with respect to the central body (Fig.2d–f, l–n).

The adult menisci showed higher GAGs deposition than the young menisci in all the different analysed areas (Fig.2).

*Double immunofluorescence*. According to the similarities observed between the medial and lateral menisci, only the medial meniscus is presented as representative for both.

**Figure 3:**
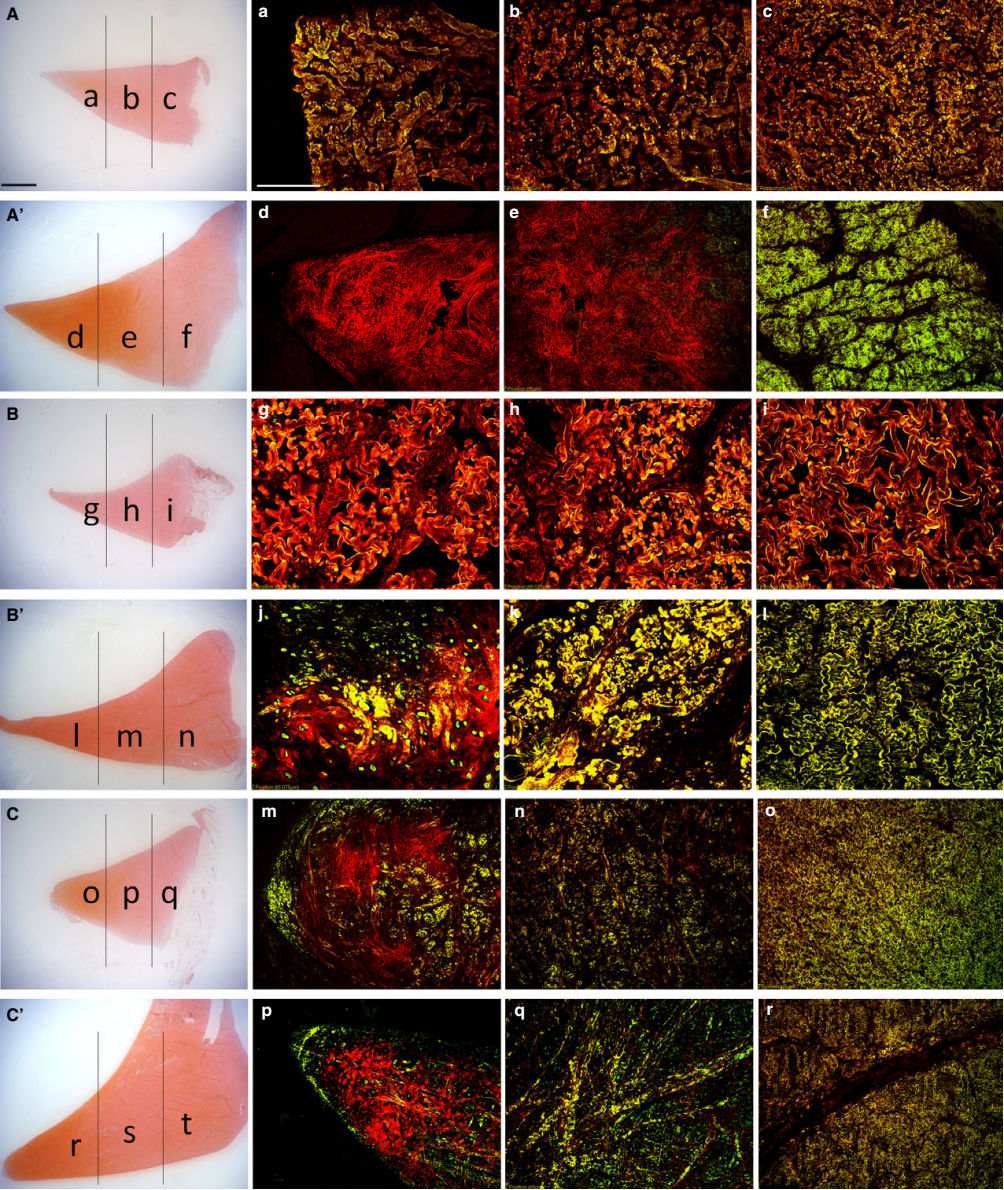
Immunofluorescent staining of collagen 1 and 2 on traversal sections: medial anterior horn of the young (**a**–**c**) and adult (**d**–**f**) meniscus; medial body of the young (**g**–**i**) and adult (**l**–**n**) meniscus; medial posterior horn of the young (**o**–**q**) and adult (**r**–**t**) meniscus. Collagen 1 is in green, collagen 2 is in red, superimposition appears in yellow colour (various degrees). Scale bar 200 μm. For comparison, two representative pictures of the young (**A**–**C**) and adult (**A**’, **B**’, **C**’) anterior horns, body and posterior horn at low magnification, stained for SAFRANIN-O. Scale bar 2000 μm. (**a**) Inner zone, anterior horn young meniscus. (**b**) Intermediate zone, anterior horn, young meniscus. (**c**) Outer zone, anterior horn, young meniscus. (**d**) Inner zone, anterior horn, adult meniscus. (**e**) Intermediate zone, anterior horn adult meniscus, (**f**) Outer zone, anterior horn, adult meniscus. (**g**) Inner zone, body, young meniscus. (**h**) Intermediate zone, body, young meniscus. (**i**) Outer zone, body, young meniscus. (**j**) Inner zone, body, adult meniscus. (**k**) Intermediate zone, body, adult meniscus. (**l**) Outer zone, body, adult meniscus, (**m**) Inner zone, posterior horn young meniscus. (**n**) Intermediate zone, posterior horn, young meniscus. (**o**) Outer zone, posterior horn, young meniscus. (**p**) Inner zone, posterior horn, adult meniscus. (**q**) Intermediate zone, posterior horn adult meniscus. (**r**) Outer zone, posterior horn, adult meniscus.

Anterior horn, young (Fig.3A): in the inner, middle and outer zone, a complete co-localization of collagen 1 and 2 in the matrix (Fig.3a–c: yellow fluorescence) was documented. Anterior horn, adult (Fig.3A’): in the inner zone, a strong immunopositivity to collagen 2 was seen (Fig.3d: red fluorescence); in the middle zone, the presence of positivity to collagen 2 (Fig.3e: red fluorescence) was noted together with a scarce collagen 1 positivity (Fig.3e: green fluorescence); in the outer zone, a complete co-localization of collagen 1 and 2 was detected (Fig.3f: yellow fluorescence).

Central body, young (Fig.3B): in the inner, middle and outer zones, a co-localization of collagen 1 and 2 immunopositivity (Fig.3g–i: yellow fluorescence) was noticed. Body, adult (Fig.3B’): in the inner zone, a immunopositivity to collagen 2 was documented in the matrix (Fig.3l: red fluorescence), but some areas of collagen 1 immunofluorescence were present (green fluorescence) as well as some areas of co-localization of both collagen 1 and 2 (yellow fluorescence); in the middle and outer zones (Fig.3m and n, respectively), an almost complete (Fig.3m) and a complete (Fig.3n) co-localization of collagen 1 and 2 immunoreactivities (yellow fluorescence) were detected.

Posterior horn, young (Fig.3C): in the inner zone, immunopositivity to collagen 2 was seen (Fig.3o: red fluorescence), in conjunction with a superficial collagen 1immunopositivity (green fluorescence) and a more deeply located co-localization for both collagen 1 and 2 (yellow fluorescence); in the middle zone, an immunopositivity to collagen 2 was evident together with an immunopositivity to both collagen 1 and 2 (Fig.3p: yellow fluorescence); in the outer zone, an almost complete co-localization for collagen 1 and 2 was evident (Fig.3q: yellow fluorescence). Posterior horn, adult (Fig.3C’): in the inner zone, a similar aspect was demonstrated with respect to the young condition (Fig.3r: red fluorescence, together with green fluorescence and yellow fluorescence); also the middle zone (Fig.3s) and the outer zone showed conditions similar to those of the young animal (Fig.3t).

#### Biochemical analyses

**Figure 4:**
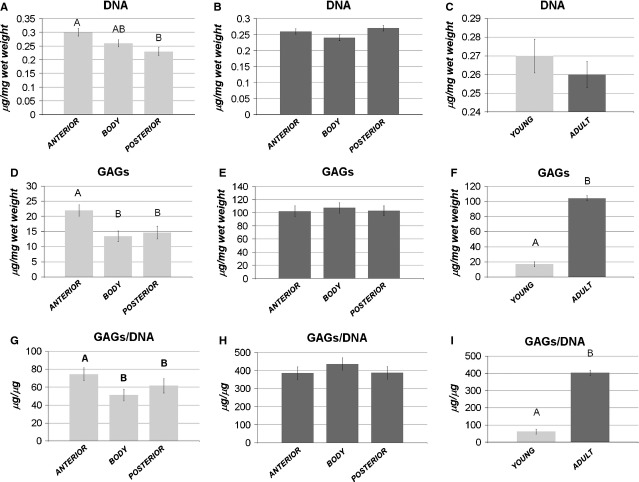
Biochemical analysis. Comparison of DNA (**A**–**B**), GAGs (**D**–**E**), and GAGs/DNA content (**G**–**H**) among the anterior horn, the body and the posterior horn: (**A**,**D**,**G**) young model; (**B**,**E**,**H**) adult model. Comparison of DNA (**C**), GAGs (**F**) and GAGs/DNA content (**I**) between the young and the adult samples. Values with different superscripts differ for *P* < 0.01 (^A,B^). N/group = 12.

*Cellularity and GAGs production*. The young menisci were characterized by a significantly higher cellularity (*P* < 0.01), GAGs deposition (*P* < 0.01) and GAGs/DNA ratio (*P* < 0.05) in the anterior horn, with respect to the body and posterior horn (Fig.4A, D and G). In the adult menisci, the horns and the central body showed a similar cellularity, GAGs and GAGs/DNA ratio with no significantly differences (Fig.4B, E and H).

Comparing young and adult menisci, GAGs and GAGs/DNA ratio appeared to be significantly higher in the adult menisci (*P* < 0.01 for both), while no difference appeared in terms of cellularity, suggesting that the cell number was maintained throughout growth, but the cells acquired a specific competence for GAGs production.

**Figure 5:**
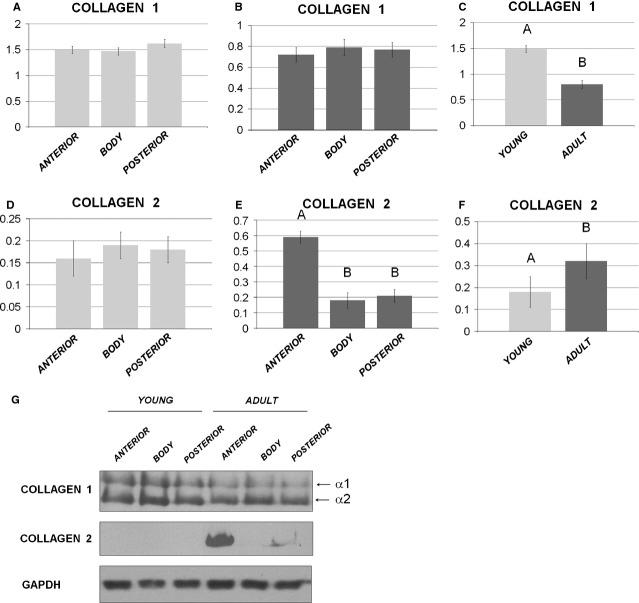
Western blot analysis. Comparison of collagen 1 (**A** and **B**) and collagen 2 (**D** and **E**) among the anterior horn, the body and the posterior horn: (**A** and **D**) young model; (**B** and **E**) adult model. Comparison of collagen 1 (**C**) and collagen 2 (**F**) content between the young and the adult specimens. Values with different superscripts differ for *P* < 0.01 (^A,B^). (**G**) Representative Western blot image for collagen 1, collagen 2 and GAPDH. N/group = 8.

*Western blot analysis*. In young animals, no quantitative differences in collagen 1 production were observed among the different meniscal areas (Fig.5A, *P* > 0.05), while collagen 2 was undetectable in all the considered areas (Fig.5G), showing only a background signal (Fig.5D, *P* > 0.05). In adult animals, collagen 1 was similar in all three analysed zones (Fig.5B, *P* > 0.05), while collagen 2 (Fig.5E) was strongly present in the anterior horn compared to both the posterior horn and the body (*P* < 0.01). Comparing young *versus* adult menisci, collagen 1 was significantly higher (*P* < 0.01) in young animals (Fig.5C), while collagen 2 was significantly higher in the menisci of adults pigs (*P* < 0.001) (Fig.5F), thus revealing an inverse relationship between the two different collagen isoforms and the acquisition of a matrix with more cartilaginous features during growth.

### Study 2

#### Gene expression analysis

**Figure 6:**
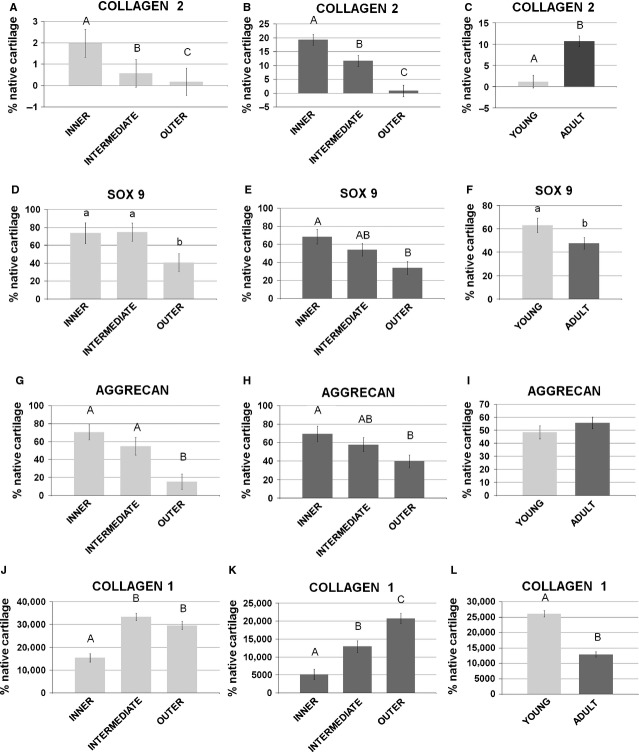
Gene expression analysis by real-time PCR. Comparison of collagen 2 (**A** and **B**), Sox9 (**D** and **E**), aggrecan (**G** and **H**) and collagen 1 (**L** and **M**) among the inner, the intermediate and the outer areas of the menisci: (**A**,**D**,**G**,**L**) young model; (**B**,**E**,**H**,**M**) adult model. Comparison of collagen 2 (**C**), Sox9 (**F**), aggrecan (**I**) and collagen 1 (**N**) between the young and the adult samples. Values with different superscripts differ for *P* < 0.05 (^a,b^) or *P* < 0.01 (^A,B^). N/group = 8.

In young pigs, collagen 2 appeared to be significantly higher in the inner zone (*P* < 0.01) with respect to intermediate and outer zones (Fig.6A); Sox9 and aggrecan were significantly higher in the inner and intermediate zones with respect to the outer one (*P* < 0.05 and 0.01 respectively; Fig.6D and G); collagen 1 resulted to be higher in the intermediate and outer zones with respect to the inner one (Fig.6L). In adult animals, collagen 2 appeared to be significantly higher in the inner zone (*P* < 0.01) with respect to the intermediate and outer zones (Fig.6B). Sox9 and aggrecan were significantly higher in the inner zone (*P* < 0.01) with respect to the outer zone, while the intermediate was similar to both the inner and outer zones (*P* > 0.05 for both) (Fig.6E and H). Comparing young *versus* adult menisci, collagen 2 appeared to be significantly higher (*P* < 0.01) in adult animals (Fig.6C), while Sox9 was significantly higher in the menisci of young pigs (*P* < 0.05; Fig.6F); aggrecan did not show statistical significance within the groups (*P* > 0.05) (Fig.6I); collagen 1 was significantly higher in the menisci of young pigs (*P* < 0.01; Fig.6N).

It is interesting to note that the expression level of Sox9 is reduced during growth (Fig.6F) with the increase in collagen 2, suggesting that this transcription factor is highly expressed only where the cartilaginous genes still have to be activated.

## Discussion

This study was aimed to highlight the changes occurring in the swine meniscus during growth. The obtained data showed that in the young and adult swine the morphology of the medial and lateral menisci resulted to be very similar in both swine models: in the young model, they both showed a scarce positivity for GAGs deposition, while in the adult specimens, they both showed a marked staining in the inner area suggesting a clear resemblance of this meniscal zone to a cartilaginous tissue. These findings are in agreement with previous description of the meniscus as a tissue having an inner proteoglycan rich matrix 24 that resembles hyaline cartilage and an external fibrous region 25–27. The matrix composition along with the anterior to posterior aspect was quantitatively characterized by GAGs measure and Western blot analysis, demonstrating an increasing production of GAGs and collagen 2 with the animal growth accompanied by a decrease in collagen 1 deposition. In particular, the acquisition of this cartilaginous component is strongly evident in the anterior horn with respect to the body and the posterior horn and is probably the result of the specific physiological mechanical stimuli that occur in the swine knee joint. The maturation of the meniscus towards a fibro-cartilaginous tissue resulted to be evident along with the medial to lateral aspect, as showed by the predominant distribution of collagen 2, with respect to collagen 1, in the inner and intermediate zones of both the horns and the body of the adult menisci. This different matrix distribution is the result of changes in the cells phenotype: these cells acquired an increased competence for the expression of cartilaginous markers and at the same time, they lost the typical fibroblasts phenotype, which is characterized by the expression of collagen 1.

Several studies characterized the complex nature of the meniscus in term of tissue composition and organization in different animal models, and many similarities were found among the human and other animal species 11,24–28. In this study, we have compared menisci from both young and adults pigs, where the young were 1-month-old animals, characterized by a reduced load-bearing activity in the knee joint, while the adults where 7-month-old animals, characterized by a higher loading pressure on the menisci. Different evidence in literature suggest that a process of maturation occurs in the meniscus in response to load increase in the knee joint, in particular for what concerns the vascular network that is strongly reduced in the adult tissue 8,29. These evidence led us to speculate that changes in meniscus composition may be a part of a re-organization programme of the meniscal tissue.

The data obtained in this study enforce the idea that the growth of the swine knee joint is accompanied by a specific fibro-chondrogenic maturation of the meniscus that occurs first posteriorly, and is then extended anteriorly, in particular, in the inner and intermediate areas.

The evidence that the meniscus architecture changes with development has been already observed by Ionescu *et al*. in bovine 12, by Bland *et al*. in rabbit 11, by Smith *et al*. 5 and Melrose *et al*. 30 in the ovine model. Our study confirms this evidence also in the swine model: we can speculate that the prevalent knee flexion that occurs in the swine model during gestation and the first weeks of life can explain the early differentiation of the posterior horn towards a fibro-cartilaginous tissue that is then followed by a marked maturation of the anterior horn in response to the increasing extension of the knee that occurs with growth. It is important to note, however, that quadrupeds are characterized by a lower extension rate of the knee joint in comparison with humans 31, and this aspect may represent a limit for the translation of the data obtained in this study to humans. Moreover, the lifespan of a pig is extremely different from that of humans: for these reason, the direct comparison between these two species presents some difficulties. However, considering the morphological variation observed by other authors in child-adult humans 8,32,33 and the same evolutionary trend observed in piglets-pig 34, we can still consider the swine models used in the present study suitable for a comparative model for humans.

The meniscus modelling and maturing during growth represents an interesting information for the development of tissue engineered strategies for partial or total meniscal substitutes. In fact, the possibility to implant an engineered meniscus with immature properties that will be further enhanced by the physiological stimuli within the knee joint, offers new alternatives such as the use of fibroblast cells or mesenchymal stem cells that are not competent for a fibro-cartilaginous matrix production, but they could become specialized upon the right mechanical stimuli. The swine meniscus has been already considered as a suitable pre-clinical model for investigating novel strategies for meniscus repair 35–37. The evidence obtained in this study may also represent a proper model for monitoring the development of such a tissue engineered meniscus and encourages the investigation of new solutions for the treatment of meniscus injuries, which may overcome the well-known limits of the surgical repair of this important structure 38, which is vital for the knee health.

## Conclusions

Overall, this study explored the developmental changes in the swine meniscus in terms of tissue morphology, matrix composition and cell phenotype. In the swine model, the meniscus is characterized by a cartilaginous competence increasing with age, occurring with a posterior to anterior direction and involving the inner and intermediate meniscal zones, and by a constant maintenance of a fibrous composition in the outer vascular zone.
